# Preadmission antidepressant use and bladder cancer: a population-based cohort study of stage at diagnosis, time to surgery, and surgical outcomes

**DOI:** 10.1186/s12885-018-4939-8

**Published:** 2018-10-24

**Authors:** Ellen Hollands Steffensen, Clint Cary, Jørgen Bjerggaard Jensen, Heidi Larsson, Michael Weiner, Mette Nørgaard

**Affiliations:** 1 Department of Clinical Epidemiology, Aarhus University Hospital, Aarhus, Denmark; 2 Department of Urology, Indiana University School of Medicine, Indianapolis, IN, USA and Regenstrief Institute, Inc, Indianapolis, IN USA; 3 Department of Urology, Aarhus University Hospital, Aarhus, Denmark and Department of Clinical Medicine, Health, Aarhus University, Aarhus, Denmark; 4 Department of Clinical Epidemiology, Aarhus University Hospital, Aarhus, Denmark and The Danish National Registries – a National Quality Improvement Programme (RKKP), Aarhus, Denmark; 5 Indiana University School of Medicine, Indianapolis, IN, USA and Regenstrief Institute, Inc, Indianapolis, IN USA; 6 Department of Clinical Epidemiology, Aarhus University Hospital, Aarhus, Denmark

**Keywords:** Urinary bladder neoplasms, Antidepressive agents, Delayed diagnosis, Cystectomy, Treatment outcome

## Abstract

**Background:**

Among cancer patients, prior antidepressant use has been associated with impaired survival. This could be due to differences in stage at diagnosis, in receipt of treatment, or in treatment complications. The purpose of this study was, therefore, to examine if preadmission antidepressant use in patients with bladder cancer is associated with tumor stage at diagnosis, rate of cystectomy, and surgical outcomes, including survival.

**Methods:**

We performed a registry-based cohort study including all patients with incident invasive bladder cancer in Denmark 2005–2015. Exposure was defined as redemption of two or more antidepressant prescriptions one year before cancer diagnosis. We compared tumor stage using logistic regression, postsurgical inpatient length of stay using linear regression, and other outcomes using Cox regression. All results were adjusted for age, sex, comorbidity, and marital status.

**Results:**

Among 10,427 bladder cancer patients, 10% were antidepressant users. At diagnosis, 51% of users and 52% of non-users had muscle-invasive disease. However, upon adjustment for age, sex, comorbidity, and marital status, users had lower odds of muscle-invasive disease (adjusted odds ratio 0.86 (95% confidence interval (CI) 0.74–0.99)). Among patients with muscle-invasive disease, fewer users than non-users had surgery within three months (15% vs. 24%, adjusted hazard ratio (aHR) 0.75 (95% CI 0.59–0.95)). Of 2532 patients undergoing surgery, 6% were antidepressant users. Postsurgical inpatient length of stay did not differ between users and non-users. The 30-day cumulative incidence of readmission was higher for users (41% vs. 33%, aHR 1.33 (95% CI 1.05–1.67)), while the 90-day incidence of postoperative procedures was 44% for users and 38% for non-users (aHR 1.18 (95% CI 0.93–1.51)). One-year mortality was comparable in users (15%) and non-users (14%).

**Conclusions:**

Antidepressant use in bladder cancer patients was associated with less advanced stage at diagnosis and lower rate of cystectomy. After cystectomy, users had higher rate of readmission and postoperative procedures than non-users, but we found no difference in length of stay or one-year mortality. The results point to the importance of differentiated clinical care according to individual patient characteristics.

**Electronic supplementary material:**

The online version of this article (10.1186/s12885-018-4939-8) contains supplementary material, which is available to authorized users.

## Background

As the ninth most commonly diagnosed cancer worldwide, bladder cancer caused a reported 165,000 deaths globally in 2012 [1]. Recommended standard treatment for muscle-invasive and high-risk non-muscle-invasive tumors is radical cystectomy, a procedure with 30-day complication rates between 24% and 73% [2]. Comorbidity raises complication risk [3], and bladder cancer patients are increasingly comorbid [4], with increased medication use including antidepressants as a consequence. The overall use of antidepressants increased by a mean of 40 defined daily doses/1000/day across Europe in the period 1980–2009 [5].

Although increasingly prescribed for neuropathic pain and sleep disorders, antidepressants are mostly prescribed for depression [6]. In a Danish setting, use of antidepressants detects patients suffering from a depression with a specificity of 94% to 97% [7]. This makes prescription data about antidepressants suited for identification of patients with depression in Denmark, where more than 90% of such patients are treated by general practitioners [7]; the treatment in these cases is not recorded in hospital registries.

In a recent Danish cohort study of patients with various types of cancer, prior antidepressant use was associated with a 30% increase in one-year mortality [8]. The study supports prior studies reporting an elevated mortality in patients with depression who developed various cancers [9–11], pancreatic cancer [12], colon cancer [13], or prostate cancer [14]. Diagnostic delay, reduced likelihood of appropriate treatment, or treatment complications may explain an increased mortality in antidepressant users. However, studies of delay in diagnosis of breast, prostate, colorectal, bladder, lung, skin, uterine, and pancreatic cancer for patients with depression show conflicting results [8–10, 12]. Prior antidepressant use was not associated with more advanced bladder cancer stage in the recent Danish work [8]. Although previous studies have reported a lower likelihood of appropriate cancer treatment in patients with prior or existing depression in a number of cancers combined [9], as well as pancreatic [12], colon [13], or prostate cancer [14], this has not been examined in patients with bladder cancer. The reported increase in one-year mortality among patients with cancer who used antidepressants before cancer diagnosis [8] is of concern and more detailed studies are needed to clarify underlying mechanisms.

Therefore, the aim of this nationwide population-based cohort study was to investigate whether antidepressant use initiated before invasive bladder cancer diagnosis was associated with more advanced cancer at time of diagnosis or time of surgery, lower rate of cystectomy, and a more complicated postoperative course reflected in length of hospital stay, readmission, postoperative procedures and mortality.

## Methods

We performed a nationwide register-based cohort study including all patients diagnosed with incident invasive bladder cancer in Denmark from 2005 through 2015. The Danish population, approximately 5.6 million inhabitants, has access to tax-supported healthcare, with free access to hospital-based and primary medical care provided by the Danish National Health Service [15]. All inhabitants in Denmark have a unique Civil Personal Register (CPR) number, which is recorded along with administrative and medical information in registries and databases and allows for person-specific linkage of information [16].

### Data sources

From the Danish National Patient Registry (DNPR), we retrieved diagnosis and procedure codes and associated dates. The DNPR has tracked all somatic hospitalizations in Denmark since 1977 and outpatient and emergency room visits to hospitals since 1995. Recorded data include CPR numbers, dates of admission and discharge, and up to 20 diagnoses, classified according to the International Classification of Diseases, tenth revision (ICD-10) since 1994 [17]. We ascertained information on tumor pathology from The Danish National Pathology Registry, which contains descriptions of pathological specimens and has a coverage of almost 100% [18]. The registry was established in 1997, and data from earlier years have been added [18]. We defined exposure status using data from the Danish National Database of Reimbursed Prescriptions [19]. This registry encompasses information on all redeemed prescriptions from 2004 onwards, including date of redemption and Anatomical Therapeutic Chemical (ATC) code. Finally, the Danish Civil Registration System provided data on marital and vital status. This database contains nearly complete, demographic data and is updated daily [16].

### Study population

We identified patients with incident, histologically verified invasive bladder cancer (thus not including patients with carcinoma in situ or pTa tumors) based on ICD-10 diagnosis codes (C67) and bladder cancer pathology (Additional file 1: Table S1). To increase the probability of truly incident cases we excluded patients fulfilling these criteria between 1995 and 2004.

For the surgical outcome analyses, we identified patients with bladder cancer and a recorded cystectomy excluding patients who received intended curative radiation therapy before cystectomy (Additional file 1: Table S1).

### Exposure, outcome measures, and covariates

We defined individuals as exposed (‘antidepressant users’) if they filled two or more antidepressant prescriptions (ATC code N06A) on separate occasions in the year preceding bladder cancer diagnosis. Using this definition we sought to reduce the number of non-adherent patients in the exposure group. Individuals filling one prescription or less are termed ‘non-users’. Patients who filled an antidepressant prescription after bladder cancer diagnosis, but not in the year before, were treated as non-users.

Stage at diagnosis was defined as muscle-invasive (pT2+) or non-muscle-invasive (pT1), and stage at cystectomy as organ confined (pT0-T2 and pN0) or non-organ confined (pT3-T4 or pN+).

We compared the cumulative incidence of cystectomy within three months following a diagnosis of muscle-invasive disease. We chose three months as a clinically relevant end of follow-up based on prior work [20]. Following surgery, we assessed outcomes expected to reflect a complicated postsurgical course: length of hospital stay, acute readmission to a somatic hospital within 30 days after discharge from the primary admission, 90-day rate of postoperative procedures, and all-cause mortality at one and three years. We defined postoperative procedures as any invasive procedure within 90 days after cystectomy (any NOMESCO Classification of Surgical Procedures code).

Based on existing literature we included as potential confounders: age, sex, marital status at diagnosis, Charlson Comorbidity Index (CCI) score [21] (excluding bladder cancer), and alcohol-related disorders. We identified comorbidities from DNPR within 10 years before cancer diagnosis (Additional file 2: Table S2) [22].

### Statistical methods

Patients’ characteristics at bladder cancer diagnosis were summarized according to exposure groups. Continuous variables were either categorized or described by their median value and interquartile range. For categorical variables, we computed proportions of individuals at each categorical level by exposure group.

We examined the association between antidepressant use and stage at diagnosis (muscle-invasive (pT2+) or non-muscle-invasive (pT1)) or surgery (organ confined (pT0-T2 and pN0) or non-organ confined (pT3-T4 or pN+)) using logistic regression, adjusting for age at diagnosis (included as an unrestricted spline with four knots in the analysis of stage at diagnosis and squared in stage at surgery), sex, CCI score (0, 1–2, 3+), alcohol-related disorders (yes/no), and marital status at diagnosis (married/not married). The analysis of stage at surgery excluded patients receiving neoadjuvant chemotherapy, which was introduced nationwide in Denmark in 2013 (Additional file 1: Table S1).

Due to missing data for tumor stage at diagnosis and at surgery, two sensitivity analyses were performed to increase the proportion of individuals with stage data: we restricted the analysis of stage at diagnosis to patients diagnosed during 2011–2015, and we repeated the analysis of stage at surgery including the latest stage recorded before surgery, if stage at surgery was missing.

For the analysis of time to surgery, we followed patients not receiving neoadjuvant chemotherapy from the date on which muscle invasiveness was first detected until cystectomy, death, emigration, end of the three months of follow-up, or 11 April 2016, whichever came first. Follow-up for postoperative procedures began at date of surgery while follow-up for readmission began at discharge. For both events, follow-up ended at event of interest, death, emigration, end of the 30-day or 90-day follow-up, or 11 April 2016, whichever came first.

We computed the cumulative incidence of cystectomy and used Cox proportional hazards regression to investigate the association between antidepressant use and cystectomy. Here, death and curative-intended radiation therapy were treated as competing risks.

We used Cox proportional hazards regression to investigate associations between antidepressant use and readmission and postoperative procedures adjusting as described for the logistic regression of stage at surgery. Death was considered competing risk. Length of hospital stay was compared between groups using a multiple linear regression model including covariates as in the logistic regression of stage at diagnosis.

For the mortality analysis, we followed patients from date of surgery until death, emigration, end of the one-year or three-year follow-up or 19 April 2016, whichever came first. We used the Kaplan-Meier estimator and compared mortality using Cox regression adjusting as described for the logistic regression of stage at surgery.

Additionally, we investigated whether neoadjuvant chemotherapy, stage at surgery, and type of surgery (open or laparoscopic and robot-assisted, (Additional file 1: Table S1)) mediated surgical outcome differences between exposure groups by addition of each of these variables individually to the regression models described above.

For all Cox regressions, the assumption of proportional hazards was assessed by log-minus-log plots.

All analyses were performed using Stata version 14 (StataCorp LP, College Station, Texas).

## Results

**Fig. 1:**
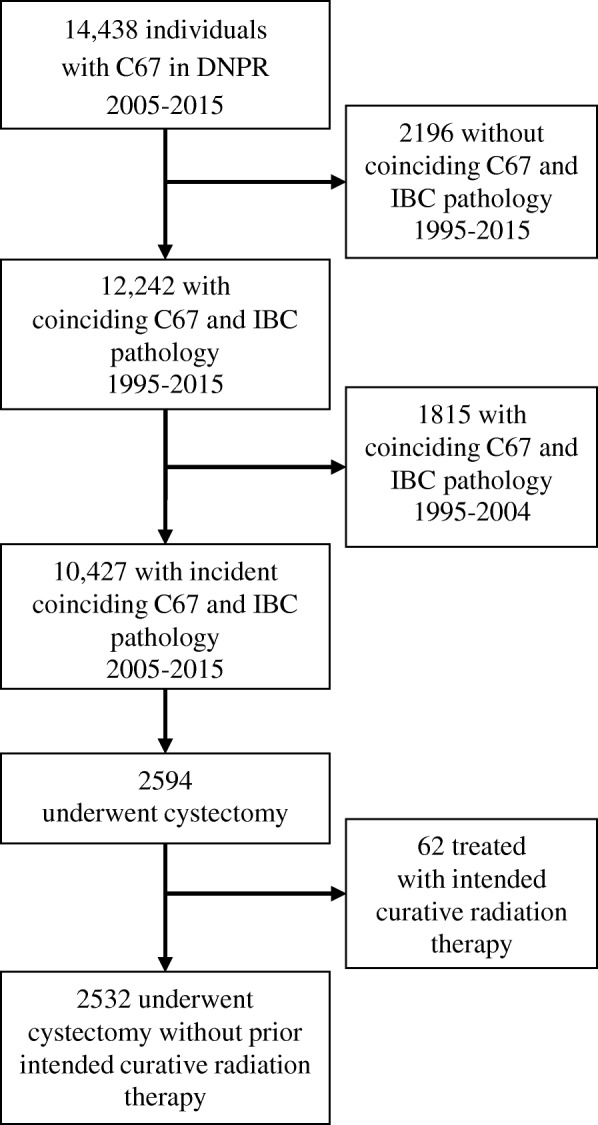
Flow diagram of the study population. DNPR: The Danish National Patient Registry. IBC: invasive bladder cancer

We identified 10,427 patients with incident invasive bladder cancer during 2005 through 2015 (Fig. 1). In the cohort, 1079 (10%) were antidepressant users. At presentation, antidepressant users were less likely to be married and had more comorbidity than non-users (Table 1).

**Table 1:** Patient characteristics at diagnosis according to use of antidepressants before cancer diagnosis

	Non-users		AD users	
	*n*	%	*n*	%
Total	9348	89.7	1079	10.3
Median age (IQR) (years)	72.5	66.0–79.8	74.4	67.8–82.3
Age group (years)				
18–49	224	2.4	22	2.0
50–59	814	8.7	85	7.9
60–69	2554	27.3	235	21.8
70–79	3475	37.2	378	35.0
80-	2281	24.4	3539	33.3
Sex				
Male	7036	75.3	647	60.0
Female	2312	24.7	432	40.0
Marital status				
Married	5549	59.4	532	49.3
Widowed	1836	19.6	320	29.7
Divorced	1233	13.2	145	13.4
Never married	730	7.8	82	7.6
Charlson Comorbidity Index score				
Low (0)	4703	50.3	334	31.0
Medium (1–2)	3124	33.4	437	40.5
High (3+)	1521	16.3	308	28.5
Alcohol-related disorders	191	2.0	68	6.3
Year of cancer diagnosis				
2005–2007	2563	27.4	271	25.1
2008–2010	2555	27.3	283	26.2
2011–2013	2527	27.0	314	29.1
2014–2015	1703	18.2	211	19.6
Stage of cancer at diagnosis				
Non-muscle-invasive (pT1)	3538	37.8	423	39.2
Muscle-invasive (pT2+)	3854	41.2	437	40.5
Missing	1956	20.9	219	20.3
Cystectomy	2370	25.4	162	15.0
Neoadjuvant chemotherapy	222	9.4	15	9.3
Intended curative radiation therapy	1145	12.3	114	10.6

All patients (*n* = 10,427) were diagnosed with incident invasive bladder cancer during 2005 through 2015. *AD* antidepressant, *IQR* interquartile range

### Stage at diagnosis and at surgery

At diagnosis, analysis of data from 79% of patients for whom pT stage data were available showed comparable proportions of muscle-invasive disease in antidepressant users and non-users, but upon adjustment, antidepressant users had lower odds of muscle-invasiveness compared with non-users (adjusted odds ratio 0.86 (95% CI 0.74–0.99)) (Table 2). Restricting the analysis to patients diagnosed during 2011 through 2015, where 93% had stage information, yielded an adjusted odds ratio of 0.87 (95% CI 0.71–1.05).

**Table 2:** Odds ratios for muscle-invasive bladder cancer at diagnosis comparing antidepressant users to non-users

	Complete case analysis						2011–2015		
	Muscle-invasive (pT2+)		Non-muscle-invasive (pT1)		Unadjusted odds ratio (95% CI)	Adjusted^a^ odds ratio (95% CI)		Unadjusted odds ratio (95% CI)	Adjusted^a^ odds ratio (95% CI)
	*n*	%	*n*	%					
Non-users (*n* = 7392)	3854	52.1	3538	47.9	Ref.	Ref.	Non-users (*n* = 3938)	Ref.	Ref.
AD users (*n* = 860)	437	50.8	423	49.2	0.95 (0.82–1.09)	0.86 (0.74–0.99)	AD users (*n* = 490)	0.96 (0.79–1.16)	0.87 (0.71–1.05)

Results of complete case analysis (2005–2015) and when restricting the analysis to 2011 through 2015. *AD* antidepressant, *CI* confidence interval. ^a^Adjusted for age, sex, CCI, alcohol-related disorders, and marital status

At cystectomy, 62% of antidepressant versus 57% of non-users had non-organ confined disease (Table 3). The adjusted odds ratio for non-organ confined disease was 1.16 (95% CI 0.78–1.74) in the 71% of patients with tumor-stage information at surgery. Additionally adjusting for time to surgery yielded an odds ratio of 1.18 (95% CI 0.79–1.77). Replacing missing values with the latest stage recorded before surgery resulted in an adjusted odds ratio of 1.19 (95% CI 0.84–1.69).

**Table 3:** Odds ratios for non-organ confined cancer at cystectomy comparing antidepressant users to non-users

	Complete case analysis						Including prior stages		
	Non-organ confined (pT3-T4 or pN+)		Organ confined (pT0-T2 and pN0)		Unadjusted odds ratio (95% CI)	Adjusted^a^ odds ratio (95% CI)		Unadjusted odds ratio (95% CI)	Adjusted^a^ odds ratio (95% CI)
	*n*	%	*n*	%					
Non-users (*n* = 1521)	859	56.5	662	43.5	Ref.	Ref.	Non-users (*n* = 2081)	Ref.	Ref.
AD users (*n* = 111)	69	62.2	42	37.8	1.27 (0.85–1.88)	1.16 (0.78–1.74)	AD users (*n* = 142)	1.29 (0.92–1.81)	1.19 (0.84–1.69)

Results of complete case analysis and when replacing missing values for cancer stage with the latest stage observed before surgery. *AD* antidepressant, *CI* confidence interval. ^a^Adjusted for age, sex, CCI, alcohol-related disorders, and marital status

### Time to surgery

**Fig. 2:**
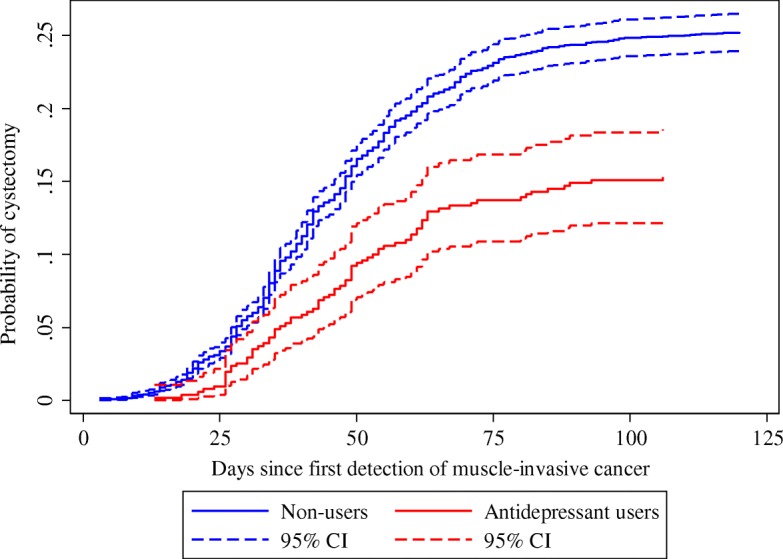
Cumulative incidence of cystectomy within three months from the first detection of muscle-invasive disease. CI: Confidence interval

We identified 4953 patients with muscle-invasive bladder cancer. The cumulative incidence of cystectomy within three months from the date on which muscle invasiveness was first detected was lower for antidepressant users than non-users (Fig. 2). The difference remained apparent after adjustment (adjusted hazard ratio 0.75 (95% CI 0.59–0.95)) (Table 4).

**Table 4:** Cumulative incidence and hazard ratio of cystectomy for patients with muscle-invasive disease comparing antidepressant users to non-users

	Cystectomized, n	Three months cumulative incidence, % (95% CI)	Unadjusted HR (95% CI)	Adjusted^a^ HR (95% CI)
Non-users (*n* = 4443)	1082	24.3 (23.1–25.6)	Ref.	Ref.
AD users (*n* = 510)	76	14.7 (11.8–18.0)	0.60 (0.48–0.76)	0.75 (0.59–0.95)

The cumulative incidence and hazard ratio of cystectomy is determined within three months from the first detection of muscle-invasive disease. *AD* antidepressant, *HR* hazard ratio, *CI* confidence interval. ^a^Adjusted for age, sex, CCI, alcohol-related disorders, and marital status

### Surgical outcomes

We identified 2532 cystectomies of which 698 (27.6%) were robot-assisted. Among cystectomized patients, 162 (6%) were antidepressant users. Table 5 presents estimates of associations between antidepressant use and surgical outcomes. Median follow up from surgery to death, loss to follow up, or end of study was 2.6 years (interquartile range 1.1 to 5.5 years).

**Table 5:** Surgical outcomes among patients undergoing radical cystectomy by antidepressant use before cancer diagnosis

Surgical outcome	Non-users	AD users
Length of stay (LOS)		
Days admitted, median (IQR)	10 (7–13)	9 (8–13)
Unadjusted ratio of median LOS (95% CI)	Ref.	1.03 (0.95–1.13)
Adjusted^a^ ratio of median LOS (95% CI)	Ref.	1.02 (0.93–1.12)
30-day acute readmission		
Total, n	2241	151
Readmitted, n	747	62
Cumulative incidence, % (95% CI)	33.4 (31.4–35.3)	41.1 (33.2–48.8)
Unadjusted HR (95% CI)	Ref.	1.36 (1.08–1.71)
Adjusted^a^ HR (95% CI)	Ref.	1.33 (1.05–1.67)
90-day postoperative procedure		
Total, n	2370	162
Procedures, n	908	72
Cumulative incidence, % (95% CI)	38.3 (36.4–40.3)	44.4 (36.7–51.9)
Unadjusted HR (95% CI)	Ref.	1.19 (0.93–1.51)
Adjusted* HR (95% CI)	Ref.	1.18 (0.93–1.51)
One-year all-cause mortality		
Total, n	2370	162
Deaths, n	330	24
Cumulative incidence, % (95% CI)	14.4 (13.1–16.0)	15.0 (10.3–21.6)
Unadjusted HR (95% CI)	Ref.	1.05 (0.69–1.58)
Adjusted^a^ HR (95% CI)	Ref.	0.96 (0.63–1.46)
Three-year all-cause mortality		
Total, n	2370	162
Deaths, n	650	52
Cumulative incidence, % (95% CI)	31.3 (29.3–33.4)	35.4 (28.1–43.8)
Unadjusted HR (95% CI)	Ref.	1.16 (0.88–1.54)
Adjusted^a^ HR (95% CI)	Ref.	1.02 (0.77–1.36)

The analysis of acute readmission excludes patients with a primary admission longer than 30 days (*n* = 140). *AD* antidepressant, *IQR* interquartile range, *CI* confidence interval, *HR* hazard ratio. ^a^Adjusted for age, sex, CCI, alcohol-related disorders, and marital status

Median duration of postsurgical stay did not differ between antidepressant users and non-users.

With a cumulative incidence of 41%, antidepressant users were more likely to be acutely readmitted within 30 days after discharge, compared with non-users (adjusted hazard ratio 1.33 (95% CI 1.05–1.67)). We observed no difference in type of department for readmission between antidepressant users and non-users (results not shown). The diagnoses related to readmission were similar among exposure groups and largely reflected postoperative complications (results not shown).

Antidepressant users’ 90-day cumulative incidence of postoperative procedures was 44% compared with 38% among non-users (adjusted hazard ratio 1.18 (95% CI 0.93–1.51)). The types of postoperative procedures were comparable among antidepressant users and non-users and mainly represented procedures related to complications to the cystectomy and urinary diversion—the five most common being: percutaneous nephrostomy, repair of wound dehiscence in urological surgery, percutaneous drainage of intraperitoneal abscess, percutaneous drainage of peritoneal cavity, and nephrostomy.

Overall one-year all-cause mortality after surgery was 14.5% (95% CI 13.1–15.9%). One- and three-year mortality showed no difference between exposure groups after adjustment.

Repeating all surgical outcome analyses additionally adjusting for neoadjuvant chemotherapy, stage at cystectomy, or type of surgery changed estimates by less than 5% (Additional file 3: Table S3).

## Discussion

In this nationwide population-based cohort study, we found that patients with bladder cancer who filled at least two antidepressant prescriptions in the year before cancer diagnosis were less likely to present with muscle-invasive disease at diagnosis (i.e. they had lower odds of muscle-invasive disease relative to non-users upon adjustment). Users also had a lower rate of cystectomy in case of muscle-invasive disease. Following surgery, antidepressant users and non-users had comparable length of stay. However, we observed a slightly higher risk of postoperative procedures within 90 days and a higher 30-day readmission rate after surgery in antidepressant users than in non-users. Still, users’ all-cause mortality was not different from non-users at one and three years.

We observed similar proportions of patients with muscle-invasive cancer at diagnosis among antidepressant users and non-users. However, this observation was likely confounded since antidepressant users had lower odds of muscle-invasive disease upon adjustment for age, sex, comorbidity, and marital status. Our result is unexpected since depression has been associated with advanced stage at cancer presentation [8, 12]. One possible explanation is an enhanced likelihood of cancer detection due to increased utilization of health care by antidepressant users [23]. Study setting may add to this: access to the tax-funded Danish health care services is free [15], minimizing the risk of patient delay in diagnosis due to patients’ financial situation or constraints. However, Sun et al. found that Danish patients with breast, colorectal, or prostate cancer who were using antidepressants presented in an advanced stage [8]. This suggests that mechanisms underlying diagnostic delay may differ between cancer types. In bladder cancer, hematuria may be so concerning to patients that it leads to cancer diagnosis even if patients are impaired by depression. In other cancers, symptoms may be less alarming, or perhaps symptoms are considered caused by depression, leading to a diagnostic delay in patients with depression who develop cancer.

Previous studies have found a lower likelihood of treatment in cancer patients with prior depression [9, 12–14]. This is in line with our finding of antidepressant users’ lower rate of cystectomy. Patient-related factors associated with decline of curative cancer treatment include patients’ perceived low quality of patient-physician communication [24], concern about side effects [25], and expected low quality of life after surgery [24] all of which may be exaggerated in antidepressant users. Another explanation could be that a larger proportion of antidepressant users than non-users received palliative care rather than radical cystectomy. We must note, however, that we did not have information on all clinical features and patient characteristics contributing to treatment decision.

We observed a high rate of postoperative procedures compared with prior studies reporting 90-day reoperation rates in the range 14–33% [2]. The discrepancy is likely explained by the fact that we included all types of procedures (not solely complication related procedures) performed 90 days after cystectomy. We note that the estimate in this analysis is somewhat imprecise, given that the 95% confidence interval includes one. Higher rates of readmission and, potentially, postoperative procedures among antidepressant users suggest a more complicated postsurgical course, while the lack of difference in length of stay speaks against any major difference. In prior studies, surgical patients with depressive symptoms had impaired wound healing and higher risk of infections [26, 27]. Our results are consistent with these findings, if antidepressant users had depressive symptoms at the time of surgery.

In contrast to prior work [8], we did not observe an association between antidepressant use and all-cause mortality in patients with cancer. Possible explanations may be that we restricted the survival analysis to patients undergoing surgery and that selection for surgery differed between antidepressant users and non-users. Antidepressant users and non-users who underwent surgery are a selected group of patients with bladder cancer. Differences in comorbidity are likely to be equalized because of the selection resulting in comparable survival. Also, Sun et al. [8] found that the increased mortality among antidepressant users depended on time of antidepressant initiation. This may contribute to the discrepancy between our studies. Another aspect to consider is severity of depression: generally, antidepressant users were probably less severely depressed than individuals included in earlier studies based on secondary psychiatric care data [10, 11], because these previous studies did not include patients with milder depression treated by general practitioners.

A strength of the current study is that by using register data, we were able to include all patients in Denmark with invasive bladder cancer during the study period. Also, we were able to control for differences in comorbidity in contrast to previous studies [9, 10]. We did not rely on self-report of exposure and we achieved essentially complete follow-up, thereby increasing generalizability. To reduce the risk of misclassification due to medication non-adherence, we required the exposed patients to have filled at least two prescriptions for antidepressants.

The study results must, however, be interpreted bearing in mind a number of limitations. Our analyses did not take into account indications for readmission and postoperative procedures. However, we only observed small differences between antidepressant users and non-users in types of postoperative procedures and readmission, and most procedure codes and readmission diagnosis codes reflected complications related to cystectomy.

We emphasize that use of antidepressants does not always indicate depression, since antidepressants are sometimes prescribed for other conditions. As such, our findings may not be directly comparable to studies investigating patients with a depression diagnosis. Also, some non-users may have suffered from a depression not treated by antidepressants. This could weaken associations between antidepressant use and outcomes such as time to cystectomy or postoperative outcomes. Prior studies based on a diagnosis of depression might have experienced a comparable misclassification of exposure due to undiagnosed depression or depression not recorded in registries [9–14].

We note that antidepressant users were generally more comorbid than non-users; a potential confounding effect we accounted for by adjusting for CCI. However, we cannot rule out the possibility of residual confounding due to e.g. comorbidity not recorded in registries. Also, we lacked information on certain additional potential confounders, such as detailed clinical characteristics, smoking, alcohol use, and socioeconomic status. A previous study, however, found no clear association between socioeconomic status and radical cystectomy [28]. Although we restricted the analysis of time to surgery to patients with muscle-invasive disease, we cannot rule out that exposure groups differed with regard to lymph node status and/or distant metastases. If more antidepressant users had distant metastases, this could explain their lower rate of cystectomy. However, antidepressant users had less advanced cancer at diagnosis, suggesting that they were not more likely to have metastases compared with non-users. Moreover, we did not observe any mortality difference between groups, which would have been expected in case of unequal proportions of patients with distant metastases. Smoking increases the risk of surgical complications [29], and may in part confound our findings on readmission and postoperative procedures. However, estimates changed very little upon adjustment for other covariates (age, sex, comorbidity, and marital status). This leads us to believe that confounding by smoking does not completely explain the observed associations. Smoking is associated with more advanced stage of bladder cancer at diagnosis [30]. Thus, if antidepressant users were more often smokers, the lower stage at diagnosis observed among them would be unexpected. On the other hand, if antidepressant users were less often smokers, our observation of no difference in mortality after surgery could be confounded by smoking. Altogether, we cannot exclude the possibility that the inability to adjust for smoking leads to residual confounding.

Our findings suggest that patients with bladder cancer using antidepressants before diagnosis may be a subgroup worth special attention—especially in relation to receipt of treatment and postsurgical care. This also points to the importance of differentiated clinical care according to individual characteristics and individual needs. For example, patients with high levels of comorbidities preoperatively, dementia, depression, etc. are subgroups who we should devote more effort to in terms of preoperative preparation and closer postoperative care following discharge. In line with this, future studies may focus on other patient subgroups and evaluate aspects in relation to receipt of treatment and treatment outcomes as such knowledge is a prerequisite for individualized clinical care.

## Conclusions

In conclusion, this nationwide population-based cohort study using Danish register data found that antidepressant use before invasive bladder cancer diagnosis was associated with less advanced stage of cancer at diagnosis. Among patients with muscle invasive disease, we observed lower rate of cystectomy in antidepressant users, and after surgery higher rate of readmission and, potentially, higher rate of postoperative procedures, but not prolonged length of stay or increased all-cause mortality. Our findings indicate that antidepressant users who develop bladder cancer may require increased medical attention.

## Additional files

Additional file 1: **Table S1.** Coding of bladder cancer, cystectomy, intended curative radiation therapy, and neoadjuvant chemotherapy. (DOCX 15 kb)

Additional file 2: **Table S2.** ICD-10 codes defining Charlson Comorbidity Index (CCI) diseases and alcohol-related disorders. (DOCX 14 kb)

Additional file 3: **Table S3.** Surgical outcomes by antidepressant use with additional adjustments. (DOCX 14 kb)

## Abbreviations

AD: Antidepressant
ATC: Anatomical Therapeutic Chemical
CCI: Charlson Comorbidity Index
CPR: Civil Personal Register
DNPR: The Danish National Patient Registry
HR: Hazard ratio
ICD-10: International Classification of Diseases, tenth revision
OR: Odds ratio
