# Supplementary material for: Preadmission antidepressant use and bladder cancer: a population-based cohort study of stage at diagnosis, time to surgery, and surgical outcomes
Source: BMC Cancer. 2018 Oct 24;18:1035. doi: 10.1186/s12885-018-4939-8 (PMC6201496; doi:10.1186/s12885-018-4939-8)
Supplement: Supplementary file 1 — Table S1. Coding of bladder cancer, cystectomy, intended curative radiation therapy, and neoadjuvant chemotherapy. (DOCX 15 kb) [file 12885_2018_4939_MOESM1_ESM.docx]

Additional file 1: Table S1. Coding of bladder cancer, cystectomy, intended curative radiation therapy, and neoadjuvant chemotherapy.

| **Criteria for identifying invasive bladder cancer** | **SNOMED codes (Danish version) in description of pathological specimen** | | **ICD-10 diagnosis codes** | |
| --- | --- | --- | --- | --- |
|  | Topography: Urinary bladder  *and*  Malignant tumor | T74  M80xx3-M84xx3 | Malignant neoplasm of bladder  *excluding*  Malignant neoplasm of urachus  Malignant neoplasm of urachus with metastases  Locally recurrent malignant neoplasm of urinary bladder | C67  C67.7  C67.7M  C67.9X |
|  | SNOMED codes must co-occur in the same pathological description and be dated within 365 days before, or 90 days after, a hospital contact with C67. Date of diagnosis is the first date of pathology in this period. | | | |
|  |  | | | |
|  |  | | | |
| **Criteria for identifying cystectomy** |  | | **NOMESCO Classification of Surgical Procedures codes** | |
|  | Open cystectomy | | KCC00, KCC10, KCC20, KCC30, KCC96 | |
|  | Laparoscopic and robot assisted cystectomy | | KCC01, KCC11, KCC21, KCC31, KCC97 (+ZXX00 for robotic procedures) | |
|  |  | |  | |
|  |  | |  | |
| **Criteria for identifying intended curative radiation therapy** | **Treatment code** | | **ICD-10 diagnosis code** | |
|  | External radiation therapy (BWGC) | | Malignant neoplasm of bladder (C67) | |
|  | At least 15 radiation treatments with C67 as indication at one of the five treatment centers in Denmark. The BWGC codes must not be preceded by a cystectomy. | | | |
|  |  | | | |
|  |  | | | |
| **Criteria for identifying neoadjuvant chemotherapy** | **Treatment code** |  | | |
|  | Cytostatic treatment (BWHA) | |  | |
|  | One to eight cytostatic treatments within 24 weeks. The last treatment must be succeeded by a cystectomy within 12 weeks. | | | |
