# Supplementary material for: Preadmission antidepressant use and bladder cancer: a population-based cohort study of stage at diagnosis, time to surgery, and surgical outcomes
Source: BMC Cancer. 2018 Oct 24;18:1035. doi: 10.1186/s12885-018-4939-8 (PMC6201496; doi:10.1186/s12885-018-4939-8)
Supplement: Supplementary file 2 — Table S2. ICD-10 codes defining Charlson Comorbidity Index (CCI) diseases and alcohol-related disorders. (DOCX 14 kb) [file 12885_2018_4939_MOESM2_ESM.docx]

Additional file 2: Table S2. ICD-10 codes defining Charlson Comorbidity Index (CCI) diseases and alcohol-related disorders.

| **CCI disease category** | **Score** | **ICD-10 code** |
| --- | --- | --- |
| Myocardial infarction | 1 | I21-23 |
| Congestive heart failure | 1 | I50, I11.0, I13.0, I13.2 |
| Peripheral vascular disease | 1 | I70-74, I77 |
| Cerebrovascular disease | 1 | I60-69, G45-46 |
| Dementia | 1 | F00-03, F05.1, G30 |
| Chronic pulmonary disease | 1 | J40-47, J60-67, J68.4, J70.1, J70.3, J84.1, J92.0, J96.1, J98.2-3 |
| Connective tissue disease | 1 | M05-06, M08-09, M30-36, D86 |
| Ulcer disease | 1 | K22.1, K25-28 |
| Mild liver disease | 1 | B18, K70.0-70.3, K70.9, K71, K73-74, K76.0 |
| Diabetes mellitus | 1 | E10.0-10.1, E10.9, E11.0-11.1, E11.9 |
| Hemiplegia | 2 | G81-82 |
| Moderate to severe renal disease | 2 | I12-13, N00-05, N07, N11, N14, N17-19, Q61 |
| Diabetes with end organ damage | 2 | E10.2-8, E11.2-11.8 |
| Any tumor | 2 | C00-26, C30-41, C43-58, C60-66, C68-75 |
| Leukemia | 2 | C91-95 |
| Lymphoma | 2 | C81-85, C88, C90, C96 |
| Moderate to severe liver disease | 3 | B15.0, B16.0, B16.2, 19.0, K70.4, K72, K76.6, I85 |
| Metastatic solid tumor | 6 | C76-80 |
| AIDS | 6 | B20-24 |
|  |  |  |
|  |  | **ICD-10 code** |
| **Alcohol-related disorders** |  | F10.1-10.9, G31.2, G62.1, G72.1, I42.6, K29.2, K86.0, Z72.1 |

AIDS: acquired immune deficiency syndrome. ICD-10: International Classification of Diseases, tenth revision.
