# Supplementary material for: Preadmission antidepressant use and bladder cancer: a population-based cohort study of stage at diagnosis, time to surgery, and surgical outcomes
Source: BMC Cancer. 2018 Oct 24;18:1035. doi: 10.1186/s12885-018-4939-8 (PMC6201496; doi:10.1186/s12885-018-4939-8)
Supplement: Supplementary file 3 — Table S3. Surgical outcomes by antidepressant use with additional adjustments. (DOCX 14 kb) [file 12885_2018_4939_MOESM3_ESM.docx]

Additional file 3: Table S3. Surgical outcomes by antidepressant use with additional adjustments.

| **Surgical outcome** | | **Non-users** | **AD users** |
| --- | --- | --- | --- |
| **Length of stay (LOS),**  **ratio of median LOS (95% CI)** | |  |  |
| Model 1 | Ref. | 1.02 (0.93-1.12) |  |
| Model 2 | Ref. | 1.02 (0.93-1.11) |  |
| Model 3 | Ref. | 1.01 (0.92-1.10) |  |
| Model 4 | Ref. | 1.01 (0.92-1.10) |  |
|  |  |  |  |
| **30-day acute readmission, HR (95% CI)** | |  |  |
| Model 1 | Ref. | 1.33 (1.05-1.67) |  |
| Model 2 | Ref. | 1.28 (0.99-1.67) |  |
| Model 3 | Ref. | 1.33 (1.02-1.74) |  |
| Model 4 | Ref. | 1.30 (1.00-1.69) |  |
|  |  |  |  |
| **90-day postoperative procedures, HR (95% CI)** | |  |  |
| Model 1 | Ref. | 1.18 (0.93-1.51) |  |
| Model 2 | Ref. | 1.18 (0.93-1.51) |  |
| Model 3 | Ref. | 1.18 (0.92-1.51) |  |
| Model 4 | Ref. | 1.19 (0.93-1.52) |  |
|  |  |  |  |
| **One-year all-cause mortality, HR (95% CI)** | |  |  |
| Model 1 | Ref. | 0.96 (0.63-1.46) |  |
| Model 2 | Ref. | 0.96 (0.63-1.47) |  |
| Model 3 | Ref. | 0.91 (0.59-1.39) |  |
| Model 4 | Ref. | 0.94 (0.62-1.44) |  |
|  |  |  |  |
| **Three-year all-cause mortality, HR (95% CI)** | |  |  |
| Model 1 | Ref. | 1.02 (0.77-1.36) |  |
| Model 2 | Ref. | 1.02 (0.77-1.36) |  |
| Model 3 | Ref. | 1.00 (0.75-1.34) |  |
| Model 4 | | Ref. | 1.01 (0.76-1.35) |

Model 1: adjusted for age, sex, CCI, alcohol-related disorders, and marital status.
Model 2: as model 1 + receipt of neoadjuvant chemotherapy.
Model 3: as model 1 + stage at surgery (non-organ confined/organ-confined).
Model 4: as model 1 + type of surgery (open/laparoscopic and robot-assisted).
AD: antidepressant. CI: confidence interval. HR: hazard ratio.
